# Berberine Regulated miR150-5p to Inhibit P2X7 Receptor, EMMPRIN and MMP-9 Expression in oxLDL Induced Macrophages

**DOI:** 10.3389/fphar.2021.639558

**Published:** 2021-04-20

**Authors:** Lin Lu, Jianjian Huang, Xia Xue, Ting Wang, Zhouqing Huang, Jianmin Li

**Affiliations:** ^1^ The Key Laboratory of Cardiovascular Disease of Wenzhou, Department of Cardiology, The First Affiliated Hospital of WenZhou Medical University, Wenzhou, China; ^2^ Department of Anesthesiology, Wenzhou Medical University, Wenzhou, China; ^3^ Department of Pathology, The First Affiliated Hospital of WenZhou Medical University, Wenzhou, China

**Keywords:** berberine, P2X7 receptor, EMMPRIN (extracellular matrix metalloproteinase inducer), MMP-9, oxLDL

## Abstract

Elevated extracellular matrix metalloproteinase inducer (EMMPRIN) and matrix metalloproteinase-9 (MMP-9) in oxidized low density lipoprotein (oxLDL)-induced macrophages leads to the progression of vulnerable plaques by degradation of the extracellular matrix. Our previous report showed that berberine regulates the expression of both EMMPRIN and MMP-9. In addition, P2X7 receptor (P2X7R) upregulation plays a crucial role in the development of atherosclerosis. However, it is unclear whether berberine regulated P2X7R level to inhibit both EMMPRIN and MMP-9 expession in macrophages. In the present study, we investigated the impact of berberine on P2X7R expression and the regulation of P2X7R in the expression of EMMPRIN and MMP-9 in oxLDL-induced macrophages. We found that P2X7R expression was increased, miR150-5p was reduced in oxLDL-induced macrophages, relatively. And A-438079 (a P2X7R inhibitor) or miR150-5p mimic treatment greatly reversed the upregulation of EMMPRIN and MMP-9 expression. Moreover, A-438079 significantly reduced oxLDL-induced AMP-activated protein kinase-α (AMPK-α) phosphorylation and reversed the activation of mitogen-activated protein kinase (MAPK), which in turn decreased the expression of EMMPRIN and MMP-9. These findings illustrate that P2X7R suppresses EMMPRIN and MMP-9 expression by inhibiting the AMPK-α/MAPK pathway in oxLDL-induced macrophages. Accordingly, exposure to berberine markedly upregulated miR150-5p, decreased P2X7R expression and downregulated MMP-9 and EMMPRIN levels in oxLDL-induced macrophages, resulting in AMPK-α/MAPK (JNK, p38, and ERK) inactivation. Overall, these results indicate that berberine increased miR150-5p level, subsequently inhibits P2X7R-mediated EMMPRIN and MMP-9 expression by suppressing AMPK-α and MAPK signaling in oxLDL-induced macrophages.

## Introduction

Atherosclerosis is a chronic and progressive inflammatory disease and is closely correlated with oxLDL-activated macrophages, which release many inflammatory factors that affect atherosclerotic plaque stability (Galis et al., 1995; Di Pietro et al., 2016; Tabas and Bornfeldt, 2016). Of particular note, the stability of atherosclerotic plaques determines whether a patient will experience cardiac events, such as acute myocardial infarction and unstable angina (Shin et al., 2003). Mounting evidence has suggested that elevated EMMPRIN and MMP-9 expression in atherosclerotic plaques (Schmidt et al., 2006) or in atherosclerosis-related cells (such as oxLDL-stimulated monocytes/macrophages and coronary smooth muscle cells) (Major et al., 2002) contributes to extracellular matrix degradation and promotes plaque rupture (Yoon et al., 2005; Joghetaei et al., 2013), which indicates that EMMPRIN and MMP-9 play important regulatory roles in the progression of atherosclerosis.

P2X purinergic receptors are adenosine triphosphate (ATP)-dependent ligand gated ion channels (Surprenant et al., 1996). Among the purinergic receptor family, P2X7 receptor (P2X7R) has a unique phenotype and is widely expressed in cardiomyocytes, monocytes, macrophages and other cells. Numerous reports have revealed that P2X7R plays an important role in different disease models, such as nervous system diseases, inflammatory bowel disease and the immune response (Stachon et al., 2017). In addition, emerging evidence indicates that P2X7R is also associated with cardiovascular disease (Chen et al., 2018). For instance, activation of P2X7R is responsible for attenuating myocardial ischemia-reperfusion injury (Vessey, et al., 2011; Chen et al., 2016). P2X7R inhibition ameliorates hypertension by reducing the inflammatory response (Ji et al., 2012; Chen et al., 2018). In atherosclerosis, P2X7R is expressed in endothelial cells and macrophages, which infiltrate the atherosclerotic plaques in human carotid arteries (Burnstock and Knight, 2004). Mantione et al. showed that P2X7R is a modulator of MMP9 secretion by primary human vascular smooth muscle-like cells and contributes to the development of atherosclerosis (Mantione et al., 2019). In addition, P2X7 deficiency attenuates atherosclerosis in LDLR−/−mice by blocking activation of NLRP3 and decreasing the release of IL-1 *β* (Peng et al., 2015; Stachon et al., 2017). However, whether P2X7R regulates EMMPRIN and MMP-9 expression in oxLDL-stimulated macrophages remains unclear.

Berberine is a natural compound that can be extracted from several medicinal herbs, such as *Cortex phellodendri* (Huangbai), *Hydrastis canadensis* and *Rhizoma coptidis* (Huanglian) (Ikram, 1975). Berberine has been extensively used in traditional Chinese medicine for many years as an anti-infectious diarrhea agent (Stermitz et al., 2000). Recently, mounting evidence has shown that berberine has pleiotropic effects in cardiovascular diseases. For example, berberine reduces plasma glucose (Zhang et al., 2018; Mi et al., 2019) and cholesterol levels (Kong et al., 2004; Cao et al., 2018). In addition, berberine inhibits excessive autophagy to alleviate cardiac ischemia-reperfusion injury in cardiomyocytes (Huang et al., 2015). Moreover, berberine exerts antiatherogenic effects by decreasing EMMPRIN and MMP-9 expression (Huang et al., 2011). However, the potential mechanisms involved in its regulation of the expression of these proteins remain elusive. Therefore, in the present study, we investigated the effects of berberine on the expression of P2X7R and the relative underlying mechanism in oxLDL-induced macrophages.

## Materials and Methods

### Cell Culture and Treatment

The human monocytic cell line THP-1 and Raw264.7 murine macrophage-like cell line was obtained from American Type Culture Collection (Rockville, MD). And THP-1 cells was maintained at a density of 10^6^/ml in RPMI 1640 medium, Raw264.7 cells were cultured in DMEM medium, containing 10% FBS, 10 mM HEPES (Sigma, United States) and 1% pen/strep solution at 37°C in a 5% CO_2_ incubator. THP-1cells were cultured in six-well plates for 48 h in the presence of 100 nM PMA, which induced differentiation into adherent macrophages (Tsuchiya et al., 1982). Then, the cells were pretreated with berberine (0–50 μM) or 100 μM A-438079 (a P2X7R inhibitor, Selleck, United States) and 10 μM compound C (AMPK inhibitor), PD98059 (a MAPK/ERK inhibitor), SB203580 (a p38 MAPK inhibitor), and SP600125 (a JNK inhibitor), which are MAP kinase inhibitors (Sigma, United States), for 1 h prior to incubation with oxLDL (50 μg/ml) for 24 h.

### MicroRNA Transfection

Cells in exponential phase of growth were plated in six-well plates at 3 * 10^5^ cells/plate and cultured overnight. Then, the cells were transfected with the miR-150 precursor (50 nM) or a negative control RNA (50 nM) using riboFECTTM CP Reagent and Buffer (RiboBio, Guangzhou, China). According to the manufacturer’s protocol of transfection reagent, the miRNAs was diluted with riboFECTTM CP buffer at 37°C for 10 min. The diluent was mixed with riboFECTTM CP Reagent and incubated for 10 min at 37°C. Then, the riboFECTTMCP-miRNA mixture was added to the cells together with 1.8 ml DMEM and incubated at 37°C for 48 h.

### Protein Isolation and Western Blot Analysis

Protein isolation and Western blot analysis of cell lysates were performed as previously described (Cao et al., 2017), except that the membranes were probed with the primary antibodies rabbit anti-GAPDH, anti-P2X7R, anti-MMP-9, anti-AMPKα, anti-phospho-AMPKα, anti-p38, anti-phospho-p38, anti-JNK, anti-phospho-JNK (Cell Signaling Technology, Boston, MA) (1:1,000 dilution in TBST), anti-EMMPRIN (Abcam, United States) (1:1,000 dilution in TBST), anti-ERK, and anti-phospho-ERK (Santa Cruz) (diluted 1:300 in TBST) at 4°C overnight. Next, the membranes were incubated with HRP-conjugated secondary antibody (1:1,000) for 1 h. Proteins were visualized by the ECL procedure (Bio-Rad, United States). The results were analyzed with Quantity One (Bio-Rad) software.

### RNA Isolation, cDNA Synthesis and Real-Time PCR

Total RNA from cells that were treated with the indicated conditions was extracted using TRIzol reagent (Invitrogen) according to the instructions. cDNA was synthesized using a cDNA transcription kit (Thermo) before real-time polymerase chain reaction (PCR) was performed using the SYBR Premix Ex Taq Kit (TaKaRa Code DRR041) as previously described (Meng et al., 2012). Real-time PCR was performed to determine the gene expression of MMP-9 and EMMPRIN. The PCR products were detected by an ABI-7500 sequence detection system (United States). The primer sequences used in the study are as follows: MMP-9 (NM_004994.2), F: TGA​CGC​CGC​TCA​CCT​TCA​CT, R: CGC​GCC​ATC​TGC​GTT​TCC​AA; EMMPRIN (NM_001728.3), Forward: TTG​GAG​GTT​GTA​GGA​CCG​GCG​A, R: TGGGACCCTGCCCTTCA- AACCA; and GAPDH (NM_001256799.2), Forward: CCG​CAT​CTT​CTT​TTG​CGT​CGC​C, R: TCT​CAG​CCT​TGA​CGG​TGC​CA; miR-150-5p (NC_000073.7), F: TCT​CCC​AAC​CCT​TGT​ACC​AGT​G, R: CAGTGCGTGTCGTGGAGT (where F is forward and R is reverse). All results were normalized to the GAPDH level.

### Gelatin Zymography

MMP activity was determined by gelatin zymography. Cells were seeded in 6-wellplates at a density of 3 × 10^5^ cells per well. After incubation in serum-free medium with or without A-438079 for 1 h, the cells were incubated with 100 μM PMA for another 48 h. Culture supernatants were collected, and 10 μL aliquots of the culture supernatant were loaded onto a 10% polyacrylamide gel containing 1 mg/ml gelatin. After electrophoresis, the gels were washed twice with 2.5% Triton X-100 (37°C, 30 min), and then the gels were incubated at 37°C for 11 h in developing buffer (10 mM Tris base, 40 mM Tris–HCl, 200 mM NaCl, 10 mM CaCl_2_, and 0.02% Brij 35). The gels were subsequently stained with 0.5% (w/v) Coomassie Blue R-250 for 2 h and then destained in the appropriate solution (50% methanol, 10% glacial acetic acid, and 40% water). MMP-9-digested regions were visualized as light bands against a dark background. An image of each gel was detected by an Odyssey imaging system (Li-cor, United States).

### Statistical Analysis

Three or more groups were compared using one-way ANOVA with Student-Newman-Keuls and Dunnett’s post hoc analysis if the ANOVA result was significant. The data are presented as the mean ± SD and were analyzed using SPSS 18 software (SPSS Inc: Chicago, IL, United States). Statistical significance was defined as *p* < 0.05. All experiments were performed at least three times.

## Results

### P2X7R Expression Was Upregulated in a Time-dependent Manner in Ox-LDL-Induced Macrophages

**FIGURE 1:**
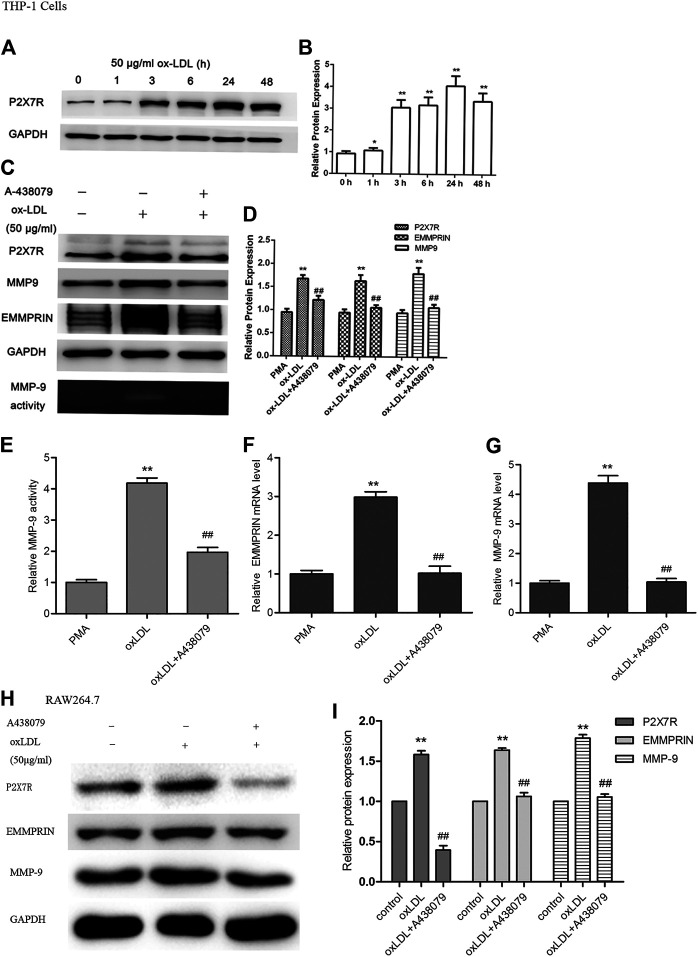
Expression of P2X7R and the effect of A-438079 on EMMPRIN and MMP-9 levels in oxLDL-induced macrophages. EMMPRIN and MMP-9 protein and mRNA levels were measured by Western blot and real-time PCR, respectively. MMP-9 activity was tested by gelatin zymography. Representative real-time PCR, Western blot and zymography results of MMP-9 or EMMPRIN expression are shown. **(A)** oxLDL induced P2X7R in a time-dependent manner in macrophages. C. Effect of A-438079 on EMMPRIN and MMP-9 levels in THP-1 cells. **(B,D,F,G)** Representative real-time PCR results of MMP-9 mRNA analysis and densitometric measurement results of EMMPRIN and MMP-9 protein levels. **(E)** Densitometric measurement results of MMP-9 activity. H and I. EMMPRIN and MMP-9 expression by A-438079 in RAW264.7 murine cells. The band densities were measured by Quantity One 1D analysis software. The data represent the mean ± S.D. *p < 0.05, **p < 0.01 compared with the PMA or control, ##*p* < 0.01 compared with the oxLDL group.

PMA-induced macrophages were treated with ox-LDL (50 μg/ml) for different times ranging from 1 to 48 h. The protein level of P2X7R was measured by Western blot analysis. We found that P2X7R expression was markedly increased by incubation with ox-LDL in a time-dependent manner (Figure 1A), and the highest level occurred at 24 h of treatment with oxLDL. Therefore, the cells were treated with 50 μg/ml ox-LDL for 24 h in subsequent experiments.

### Inhibition of P2X7R Reversed MMP-9 and EMMPRIN Expression and Decreased MMP-9 Activity in Ox-LDL-Induced Macrophages

To determine the effect of P2X7R on the expression of EMMPRIN and MMP-9, macrophages were pretreated with A-438079 for 1 h and then incubated with 50 μg/ml ox-LDL for 24 h. We showed that EMMPRIN and MMP-9 expression obviously increased after stimulation with ox-LDL, and suppression of P2X7R expression by A-438079 significantly inhibited the ox-LDL-induced upregulation of MMP-9 and EMMPRIN (Figures 1C,H), and their relative mRNA level represented the same tendency (Figures 1F,G). In addition, MMP-9 is reported to greatly enhance elastin degradation *in vitro* and induce plaque rupture *in vivo* (Galis et al., 1994; Gough et al., 2006), and we further investigated the effect of A-438079 on MMP-9 enzymatic activity. We also observed that the elevated MMP-9 activity was abolished by A-438079 (Figures 1C,E). These data indicate that P2X7R inhibition decreases EMMPRIN and MMP-9 levels and reduces MMP-9 activity in ox-LDL-stimulated macrophages.

### P2X7R Inhibition Suppressed Activation of AMPK and MAPK Pathways Induced by Ox-LDL in Macrophages

**FIGURE 2:**
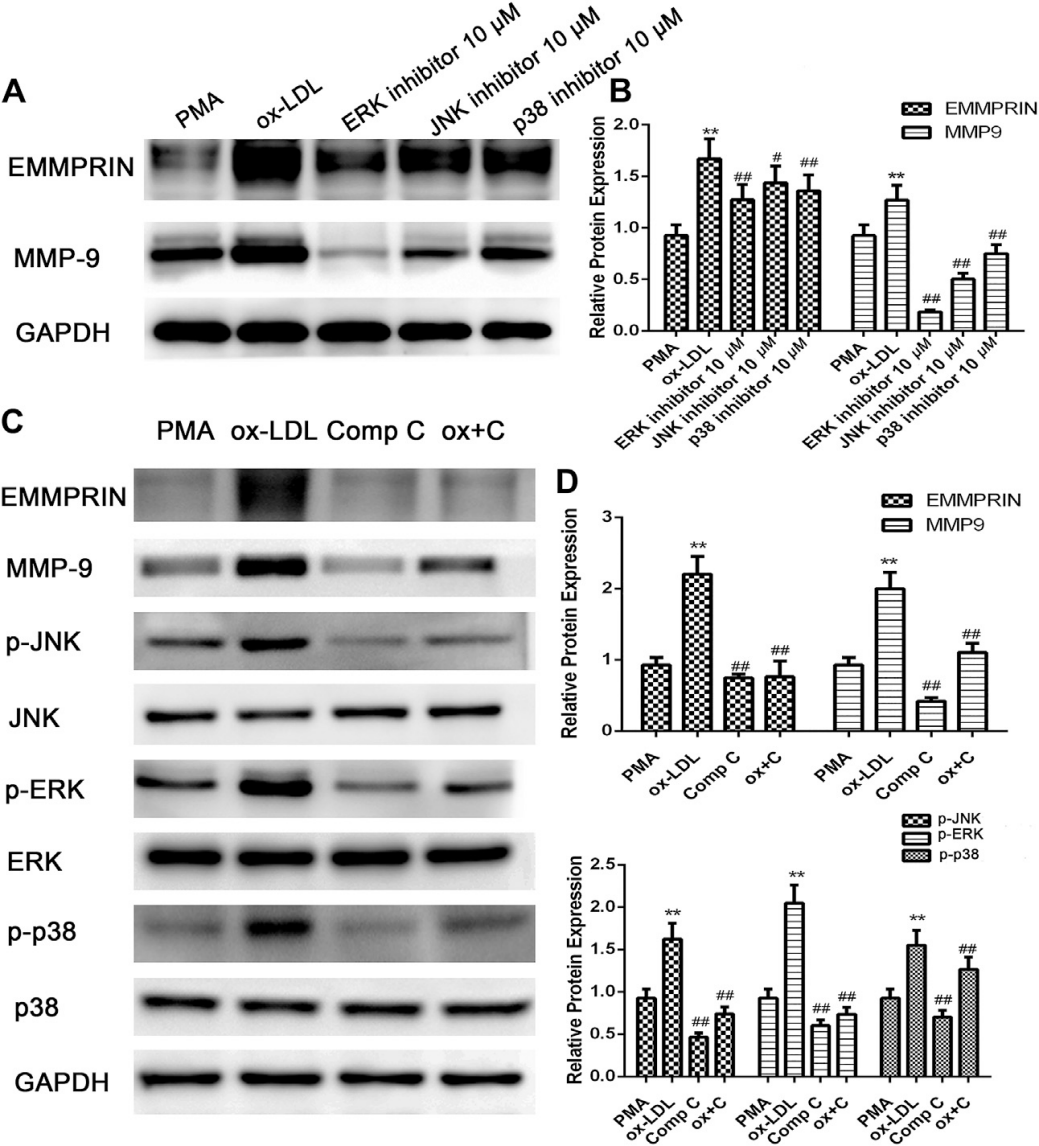
oxLDL-induced EMMPRIN and MMP-9 expression in macrophages is abolished by specific ERK, JNK, and p38 inhibitors or compound C. EMMPRIN and MMP-9 protein levels or phosphorylation of ERK1/2, p38, and JNK were measured by Western blotting. Densitometry measurements of phosphorylated ERK1/2, p38, and JNK were normalized to total ERK1/2, p38, and JNK, respectively. The band densities were measured by Quantity One 1D analysis software. The Data (means ± SD) were obtained from three independent experiments. **p < 0.01 compared with PMA, #*p* < 0.05, ##: *p* < 0.01 compared with the oxLDL group.

We previously reported (Cao et al., 2014; Cao et al., 2017) that activation of the MAPK (including ERK, p38 and JNK) or AMPK pathway is involved in the regulation of EMMPRIN and MMP-9 expression in PMA- or oxLDL-induced macrophages. In the present study, we reconfirmed the results of MAPK pathway activation in oxLDL-induced macrophages using ERK-, p38- or JNK-related inhibitors (Figure 2A). Then, to further elucidate whether AMPK has an effect on the MAPK pathway after cells are exposed to oxLDL, we used compound C to inhibit the AMPK pathway. As shown in Figure 2C, we found that compound C suppressed the phosphorylation of ERK, p38, and JNK and dramatically reduced the expression of EMMPRIN and MMP-9 in macrophages, suggesting that the AMPK inhibitor diminished activation of the p38, JNK, and ERK pathways. Thus, AMPK activation, as an upstream pathway of MAPK, is important for oxLDL-induced MMP-9 and EMMPRIN expression in macrophages.

**FIGURE 3:**
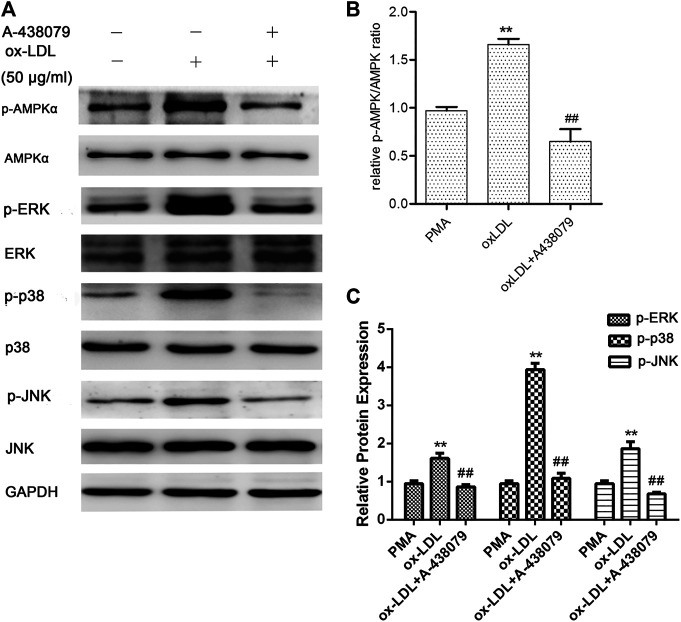
Effect of A-438079 on the phosphorylation of AMPKα, ERK1/2, p38, and JNK. The phosphorylation of AMPKα, ERK1/2, p38, and JNK was measured by Western blotting. Densitometry measurements of phosphorylated AMPKα, ERK1/2, p38, and JNK were normalized to total AMPKα, ERK1/2, p38, and JNK, respectively. The band densities were measured by Quantity One 1D analysis software. The data (means ± SD) were obtained from three independent experiments. **p < 0.01 compared with PMA, ##: *p* < 0.01 compared with the oxLDL group.

In addition, to investigate the role of P2X7R in the activation of AMPK and the MAPK pathway, we pretreated macrophages with A-438079 for 1 h and then induced them with ox-LDL for another 24 h. The phosphorylation and total protein levels of AMPK-α, ERK1/2, p38 MAPK, and JNK were examined by Western blot analysis. As shown in Figure 3, ox-LDL induced sustained activation of AMPKα and increased phosphorylation of ERK, p38, and JNK in macrophages, and these effects were markedly abolished by A-438079. Taken together, we concluded that P2X7R inhibition remarkably abolished AMPK-α phosphorylation through MAPK pathway activation, which led to decreased EMMPRIN and MMP-9 expression in oxLDL-induced macrophages.

### Berberine Reduced P2X7R Expression and Decreased EMMPRIN and MMP-9 in a Dose-dependent Manner by Inhibiting AMPK-α Though the MAPK Pathway in oxLDL-Induced Macrophages

**FIGURE 4:**
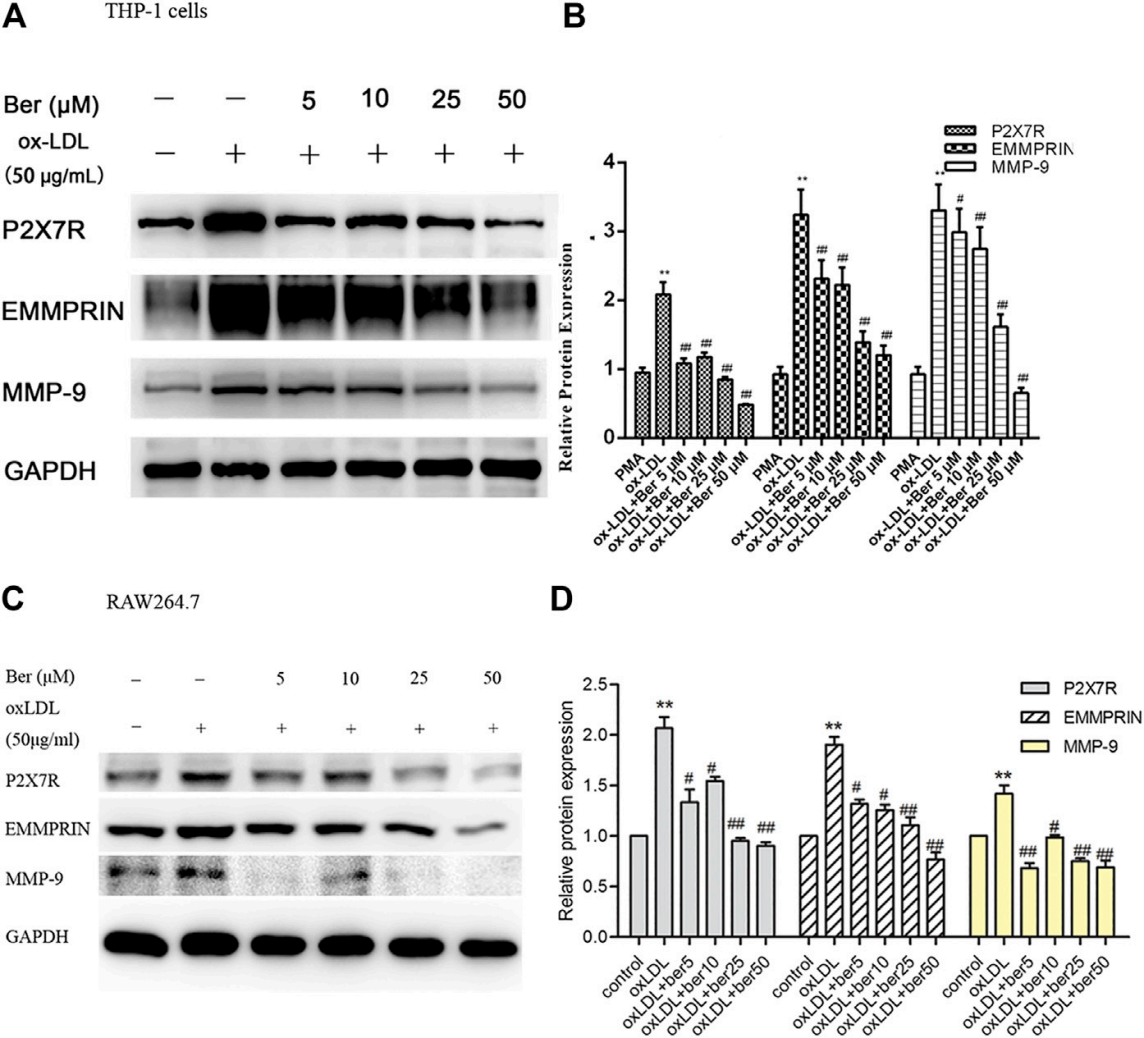
Effect of berberine (5–50 μM) on P2X7R, EMMPRIN, and MMP-9 expression **(A)** and **(B)** in THP-1 cells, **(C)** and **(D)** in RAW264.7 cells). Densitometry measurements of P2X7R, EMMPRIN, and MMP-9 were normalized to GAPDH. The band densities were measured by Quantity One 1D analysis software. The data (means ± SD) were obtained from three independent experiments. **p < 0.01 compared with PMA or control, #: *p* < 0.05, ##: *p* < 0.01 compared with the oxLDL group.

**FIGURE 5:**
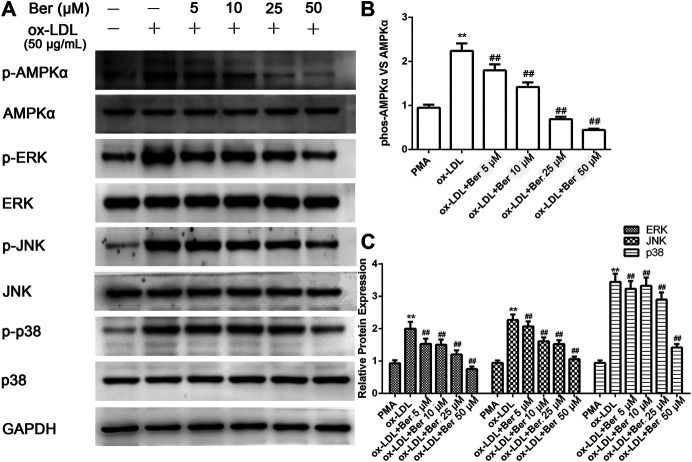
Berberine inhibited the phosphorylation of AMPKα, ERK1/2, p38, and JNK in oxLDL-induced macrophages. The densitometry measurements of phosphorylated AMPKα, ERK1/2, p38, and JNK were normalized to total AMPKα, ERK1/2, p38, and JNK, respectively. The band densities were measured by Quantity One 1D analysis software. The data (means ± SD) were obtained from three independent experiments. **p < 0.01 compared with PMA, ##: *p* < 0.01 compared with the oxLDL group.

Elevated MMP-9 and EMMPRIN expression is reduced by berberine in PMA- or oxLDL-induced macrophages (Huang et al., 2011; Huang et al., 2012), and P2X7R inhibition decreased MMP-9 and EMMPRIN expression in this study. We next tested whether the inhibitory effect of berberine on EMMPRIN and MMP-9 expression was due to P2X7R suppression in oxLDL-induced macrophages (THP-1 cells and RAW264.7). As expected, berberine reduced P2X7R levels in a dose-dependent manner while also reducing EMMPRIN and MMP-9 expression (Figure 4). Thus, downregulation of P2X7R by berberine is responsible for the reduction in EMMPRIN and MMP-9 expression in oxLDL-induced macrophages. Notably, berberine also obviously inhibited the activation of AMPKα and reduced the phosphorylation of ERK, p38 and JNK in macrophages (Figure 5), suggesting that the AMPKα and MAPK pathways induced by berberine contribute to the downregulation of P2X7R, EMMPRIN, and MMP-9 expression in oxLDL-induced macrophages.

### Berberine Increased miR-150-5p, Subsequently Repressed P2X7R Expression as Well as EMMPRIN and MMP-9 in oxLDL-Induced Macrophages

To investigate the underlying mechaniam of berberine on P2X7R.

**FIGURE 6:**
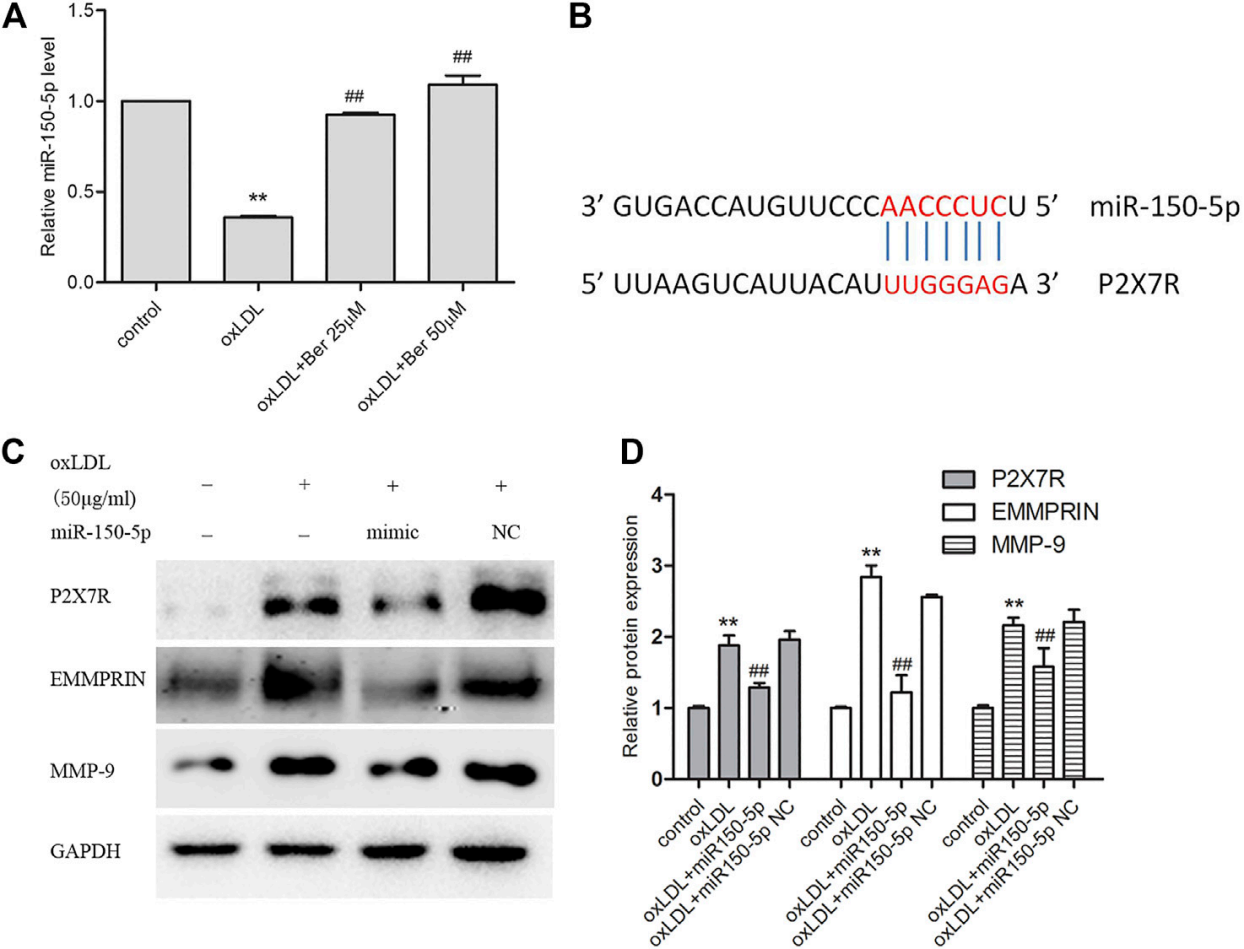
Berbeine upregulated miR-150-5p expression **(A)**; P2X7R and miR-150 have strong binding sites at their 3′-UTRs. MiR-150 seed pairing in the target regions P2X7R is shown as red vertical lines **(B)**; **(C)** overexpression miR-150-5p repressed P2X7R as well as EMMPRIN, MMP-9. Densitometry measurements of P2X7R, EMMPRIN, and MMP-9 were normalized to GAPDH. The band densities were measured by Quantity One 1D analysis software. The data (means ± SD) were obtained from three independent experiments. **p < 0.01 compared with Control, #: *p* < 0.05, ##: *p* < 0.01 compared with the oxLDL group.

Figure 6A depicted the change of miR-150-5p (mature of miR-150) after oxLDL stimulation in macrophages. We observed that miR-150-5p was reduced in oxLDL-induced macrophages, and abolished by berberine. In order to identify the relationship between miR-150 and P2X7R, we used several prediction algorithms including miRDB and Targetscan, which indicated that P2X7R is a candidate target of miR-150. The binding sites of miR-150-5p were well conserved between P2X7R mRNAs from mouse and human. As showed in Figure 6B, P2X7R has strong miR-150 binding sites at the 3′-UTR. In addition, P2X7R as well as EMMPRIN and MMP-9 were inhibited with miR-150-5p overexpression (Figure 6C).

## Discussion

In the present study, we first revealed that P2X7R expression was markedly increased in oxLDL-stimulated macrophages, and the upregulation of EMMPRIN and MMP-9 was greatly reversed by A-438079 to inhibit P2X7R. In addition, exposure to berberine markedly increased miR-150-5p, decreased P2X7R expression and downregulated the expression of MMP-9 and EMMPRIN in oxLDL-stimulated macrophages. And miR-150-5p overexpression also directly inhibited all of P2X7R, EMMPRIN, and MMP-9, suggesting that berberine inhibited P2X7R by upregulating miR-150-5p to reduce EMMPRIN and MMP9 expression in oxLDL-stimulated macrophages.

It has been recognized that the primary cause of acute myocardial syndromes is coronary artery plaque vulnerability and rupture, which lacks a collagen extracellular matrix. A growing body of evidence now indicates that overproduction of EMMPRIN and MMP-9 is critical in the degradation of the extracellular matrix (Newby, 2008; Seizer et al., 2010; Canyelles et al., 2018). MMP-9 yields caspase-1-independent activation of the proinflammatory cytokine interleukin 1 beta (IL-1β) (Mandal et al., 2003), which is a gatekeeper of inflammation; in turn, IL-1β produced by plaque endothelium, smooth muscle cells, and monocytes/macrophages increased the expression and activity of MMP 9 (Du et al., 2019). As an upstream regulator of MMP-9, EMMPRIN is also closely correlated with the progression of atherosclerosis (Schmidt et al., 2006). Consequently, inhibiting EMMPRIN and MMP-9 levels can be beneficial for the stability of atherosclerotic plaques. Notably, we previously revealed that P2X7R effectively regulates both EMMPRIN and MMP-9 during monocyte differentiation into macrophages (Lin et al., 2018). In this study, we demonstrated that P2X7R was upregulated under oxLDL stimulation in macrophages and that its inhibition significantly reduced EMMPRIN and MMP-9 expression, which indicates that P2X7R is a regulator of EMMPRIN and MMP-9 and could be a potential therapeutic target for inhibiting plaque rupture or retarding atherosclerosis. As was previously reported, the common P2X7R missense variant reduces the risk of atherosclerosis (Gidlof et al., 2012). In addition, P2X7R activation is strongly correlated with lipid accumulation in high-fat diet mice (Asatryan et al., 2015). Apart from this, atherosclerotic lesional macrophages are the major source of P2X7 receptors, and activation of P2X7R triggers an inflammatory response by activating NLRP3 and increasing IL-1β and MMP-9 (Lombardi et al., 2017; Mantione et al., 2019). These results support the concept that P2X7R plays a critical role in atherosclerosis. Accordingly, knockout of P2X7R decreases the number of lesional macrophages, resulting in a smaller atherosclerotic lesion (Stachon et al., 2017), and inhibition of P2X7R may reduce lipid oxidation, leading to the improvement of atherosclerosis (Guha et al., 2013). Thus, P2X7R is involved in the atherosclerotic process through different mechanisms, and P2X7R inhibition is beneficial for retarding the progression of atherosclerosis.

Interestingly, berberine effectively reduced the expression of P2X7R and decreased both EMMPRIN and MMP-9 in a dose-dependent manner in oxLDL-induced macrophages, suggesting that berberine inhibits EMMPRIN and MMP-9 levels by downregulating P2X7R. It is notably, the potential mechanism of how berberine regulates P2X7R in oxLDL-macrophages remains unclear. According to the data of candidate prediction and previous evidences indicated P2X7R was found to be regulated by miR-150 in pulmonary cells and HL-1 cells (Zhou et al., 2008; Weng et al., 2012; Yao et al., 2015), we tested the miR-150-5p level in oxLDL-macrophages. As expected, we observed that miR-150-5p was reduced by oxLDL and reversed by berberine. Apart from this, miR-150-5p overexpression greatly repressed P2X7R, EMMPRIN and MMP-9 expression. Collectively, the data supported that berberine suppressed P2X7R by upregulating miR-150-5p in oxLDL-macrophages.

Previous studies (Huang et al., 2008; Huang et al., 2011; Cao et al., 2014; Liang et al., 2016) reported that activation of AMPK and MAPK cascades (p38, ERK1/2, and JNK) are implicated in inducing the expression of EMMPRIN and MMP-9 in PMA-induced THP-1 cells. Here, we also tested both the AMPK and MAPK pathways in macrophages under oxLDL stimulation. As expected, oxLDL induced activation of AMPKα and increased the phosphorylation of p38, ERK1/2, and JNK in macrophages. Moreover, P2X7R inhibition using A-438079 obviously abolished both activation of AMPKα and the MAPK pathway. In addition, compound C also diminished the phosphorylation of MAPK signaling (ERK, JNK, and p38), suggesting that AMPKα is an upstream gene of MAPK activation and regulates EMMPRIN and MMP-9 expression in oxLDL-induced macrophages. Taken together, P2X7R inhibition reduced EMMPRIN and MMP-9 by reducing AMPKα inactivation through suppressing MAPK pathways. In addition, we observed the same trend in the effect of berberine on AMPKα and MAPK activation by oxLDL. These findings revealed that berberine decreases P2X7R levels, which inhibits MMP-9 and EMMPRIN expression by inhibiting AMPKα though MAPK in oxLDL-induced macrophages.

## Data Availability

The original contributions presented in the study are included in the article/Supplementary Material, further inquiries can be directed to the corresponding authors.
